# Prospectus of the World’s Columbian Dental Congress

**Published:** 1893-03

**Authors:** 


### EDITORIAL.

### The Prospectus of the World's Columbian Dental Congress, to be held in Chicago, August 14th to 19th, 1893.

This from the General Executive Committee of the Congress, through its Secretary, Dr. A. O. Hunt, of Iowa City, has just been issued, and consists of 44 pages of closely-printed matter. It gives the entire organization of the Congress so far as it has been accomplished. It gives the articles of organization and a full list of committees and officers not only of the United States, but of those of all foreign countries; the constitution and rules are also given. The duties of the various committees are fully defined.

If the Congress is not a success it will not be for the want of thorough organization; much time has been devoted by the General Executive Committee in effecting this organization, and the indications are that the Congress will be a success far beyond anything of the kind that has ever been attempted. A great deal of work is being done in the profession for the Congress, and an almost universal interest is manifested in its behalf. The General Executive Committee have held five sessions, embracing from two to three days each, and have devoted a large amount of earnest labor to the work. This undoubtedly will be the largest gathering of dentists the world has ever seen, and its equal will not be again witnessed, at least, by this generation.

